# Spectral Imaging to Measure Heterogeneity in Membrane Lipid Packing

**DOI:** 10.1002/cphc.201402794

**Published:** 2015-03-05

**Authors:** Erdinc Sezgin, Dominic Waithe, Jorge Bernardino de la Serna, Christian Eggeling

**Affiliations:** [a] MRC Human Immunology Unit and Wolfson Imaging Centre Oxford, Weatherall Institute of Molecular Medicine, University of Oxford Headley Way, Oxford, OX3 9DS (United Kingdom) E-mail: erdinc.sezgin@rdm.ox.ac.uk christian.eggeling@rdm.ox.ac.uk

**Keywords:** C-laurdan, generalized polarization, lipid packing, membrane rafts, spectral imaging

## Abstract

Physicochemical properties of the plasma membrane have been shown to play an important role in cellular functionality. Among those properties, the molecular order of the lipids, or the lipid packing, is of high importance. Changes in lipid packing are believed to compartmentalize cellular signaling by initiating coalescence and conformational changes of proteins. A common way to infer membrane lipid packing is by using membrane-embedded polarity-sensitive dyes, whose emission spectrum is dependent on the molecular order of the immediate membrane environment. Here, we report on an improved determination of such spectral shifts in the emission spectrum of the polarity-sensitive dyes. This improvement is based on the use of spectral imaging on a scanning confocal fluorescence microscope in combination with an improved analysis, which considers the whole emission spectrum instead of just single wavelength ranges. Using this approach and the polarity-sensitive dyes C-Laurdan or Di-4-ANEPPDHQ, we were able to image—with high accuracy—minute differences in the lipid packing of model and cellular membranes.

## 1. Introduction

The bioactivity of the cellular plasma membrane is correlated to membrane topology and local variations in lipid–protein compositions.[1–2] Consequently, cellular signalling is often accompanied by molecular re-organization at the membrane, and the role of physicochemical parameters in this is challenged. In particular, changes in the lateral density and order of the lipids (denoted lipid packing) are regarded to play a pivotal role, since these may introduce coalescence and conformational changes of proteins, and thus compartmentalization of cellular signaling.[3] Membrane-embedded fluorescent molecules such as Laurdan[4] and Di-4-ANEPPDHQ[5–6] are employed to report on the relative levels of membrane lipid packing.[7] The fluorescence emission of these reporters shifts depending on the order of the immediate membrane environment. In the case of Laurdan, the emission spectrum is characteristic for water dipolar relaxation processes (i.e. the water content) in the vicinity of the probe, which is a property sensitive to the membrane lateral packing.[8] Parasassi et al[4, 9–10] determined a large red shift in fluorescence emission of Laurdan when comparing its emission spectrum between a single component gel-like and a liquid-crystalline lipid model membrane. The most extensively used measure for relative levels of lipid packing is the generalized polarization (GP) parameter, whose value is a relative index of lipid packing based on red- or blue-shifted emission of the aforementioned probes.[9–11] Initially, GP was thoroughly studied employing the whole set of information derived from both the excitation and emission spectra.[9] Thereafter, to simplify the calculation, GP generally has been calculated by using the fluorescence intensities detected for two specific wavelengths. These wavelengths are usually chosen as the wavelengths *λ*_Ld_ and *λ*_Lo_ of maximum emission of the probe in a reference liquid-disordered (Ld) and liquid-ordered (Lo) membrane environment, respectively. For Laurdan, these are, for example, *λ*_Ld_=490 nm (red-shifted) and *λ*_Lo_=440 nm (blue-shifted).[12] C-Laurdan, as used in this study, is a brighter and more sensitive derivative of Laurdan, which is based on the same principle and works in the same wavelength region.[13] For Laurdan or C-Laurdan, the GP values are calculated by using the fluorescence signal intensities *I*_R_ and *I*_B_ at the red- and blue-shifted emission wavelengths *λ*_Ld_ and *λ*_Lo_, respectively [Eq. (1)]:


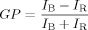
(1)

The *GP* values for other membrane-sensitive dyes can be calculated in a similar way, with *λ*_Ld_ and *λ*_Lo_ defined at different wavelengths.[7, 14] Usually, the spectra are precisely recorded on a fluorescence spectrophotometer.[4] Such measurements, however, only give values averaged over the whole sample. In many cases, such as when observing living cells, it is desirable to determine the spatial heterogeneity of the GP values, that is, to image the sample and calculate the GP at every image pixel.[15] Usually, this is achieved by recording the detected fluorescence emission in two discrete wavelength ranges centered around *λ*_Ld_ and *λ*_Lo_, for example, using two detectors and band-pass filters.[6, 12, 15–16]

**Figure 1:**
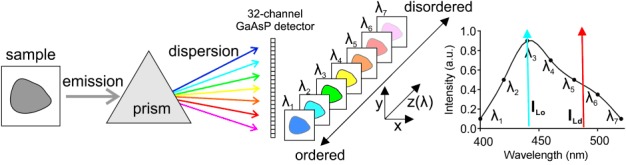
Spectral GP imaging: Fluorescence emission from the sample is generated by illumination with laser light. The collected fluorescence light is dispersed (using a prism or other device) and guided onto several parallel detectors, such as a sensitive 32-channel GaAsP detector. Each channel of the detector records a signal at different wavelengths at (in our case) approximately 8.9 nm wavelength intervals. This signal is then used to generate the emission spectrum of the fluorophore for each image pixel (only 7 of the 32 channels are shown), giving precise values of the intensities *I*_B_ and *I*_R_ (blue and red arrows).

Here, we describe an accurate method for observing spatial heterogeneity in lipid packing or GP values by recording the whole emission spectrum for each image pixel. In such a spectral (or lambda) imaging mode, a confocal scanning microscope is equipped with a diffraction grating, prism or acoustic–optical element, which spectrally disperses the collected fluorescence emission into multiple detection channels on, for example, an array of gallium arsenide phosphide (GaAsP) detectors (in our case 32-channel). Thus, it is possible to simultaneously record images over a wide range of distinct wavelengths, and to generate emission spectra for each image pixel with <10 nm spectral accuracy (Figure 1). Using a custom Fiji/ImageJ plug-in, we demonstrate an improved determination of GP values by modeling the spectral data at each image pixel using a Gaussian or Gamma Variate distribution. This allows us to precisely observe differences in lipid packing over space of phase-separated giant unilamellar vesicles (GUVs) or cell-derived giant plasma membrane vesicles (GPMVs),[17] and cellular plasma membranes following cholesterol depletion. Spectral or lambda imaging modes are realized on most recent confocal microscopes. Consequently, the use of state-of-the-art confocal systems together with the presented advanced calculation of GP values using the provided Fiji/ImageJ plug-in (curve fitting of the full spectra) is straightforward and has the potential to gain accurate insights into membrane heterogeneity and bioactivity. Our approach is therefore more general compared to a recently published approach, which is based on phasor analysis of spectral images recorded for Laurdan on a two-photon microscope.[18]

## 2. Results and Discussion

### 2.1. Spectral GP Imaging of Model Membranes

We first exemplified GP spectral imaging on model membranes such as giant unilamellar vesicles (GUVs) composed of different mixtures of lipids. We have chosen C-Laurdan[13, 19] as the environment-sensitive probe due to its common use and its increased fluorescence brightness and increased sensitivity in GP determination.[12–13, 20] The equatorial planes of the vesicles doped with C-Laurdan were imaged using a confocal microscope.[19] The fluorescence emission of C-Laurdan was excited using a 405 nm laser and detected between 415 and 700 nm by a 32-channel GaAsP detector at ≈8.9 nm wavelength intervals.

**Figure 2:**
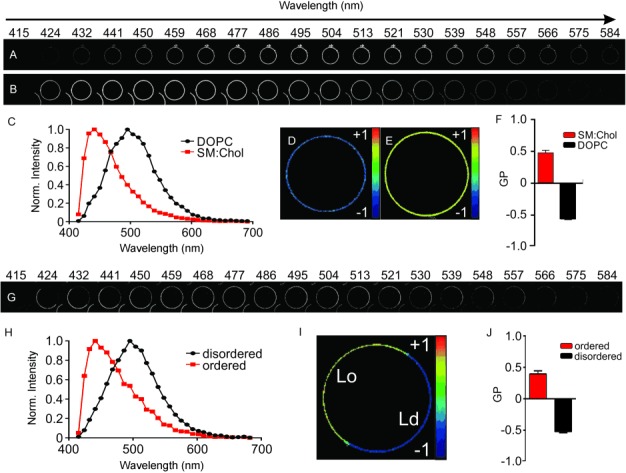
Spectral GP imaging of GUVs. A–F) Representative single-phase vesicles. A,B) Simultaneously recorded 30 μm x 30 μm large fluorescence images for 20 different 8.9 nm-wide spectral wavelength windows between 415 and 584 nm of the equatorial plane of representative DOPC (A) and SM:Chol (B) GUVs labeled with the environment-sensitive dye C-Laurdan. Notice the red-shift in fluorescence for the more disordered DOPC GUVs. C) Representative fluorescence emission spectra of C-Laurdan in a single pixel of the recorded image stacks for the DOPC (black) and SM:Chol (red) GUVs. D,E) Final GP image (size 30 μm x 30 μm) of the DOPC (D) and SM:Chol (E) GUVs. F) Average and standard deviation of the GP values determined from the GP images recorded for at least 5 GUVs. G–J) Representative phase-separated vesicles. G) Simultaneously recorded 30 μm x 30 μm large fluorescence images (as in A,B) of the equatorial plane of representative phase-separated DOPC:SM:Chol (2:2:1) GUVs labeled with the environment-sensitive dye C-Laurdan. Notice the shift in fluorescence for the different parts of the GUVs. H) Representative fluorescence emission spectra of C-Laurdan in a single pixel of the recorded image stacks for the disordered (black, more red-shifted fluorescence) and ordered (red) phases. I) Final GP image (size 30 μm x 30 μm) of the GUV. J) Average and standard deviation of the GP values determined from the pixels of the disordered and ordered regions of the images recorded for at least five GUVs.

Figures 2 A and 2 B show 20 (of the 32) image slices recorded for the equatorial plane of representative single-component dioleoyl-sn-glycero-3-phosphatidylcholine (DOPC) and two-component SM:Chol [saturated sphingomyelin (SM) and cholesterol (1:1) mixture] GUVs, respectively. Each slice exposes the spatially resolved fluorescence signal detected at 20 (of the 32) different wavelength ranges between 415 and 584 nm, and all 32 image slices allow generating emission spectra for each image pixel (Figure 2 C). It is worth noting that we miss a small fraction of the C-Laurdan spectrum (between 395–415 nm) by using a conventional microscope equipped with a 405 nm laser as the most bluish laser and a detector with a cut-off at 415 nm. However, this missed fraction does not influence our GP analysis, since the first point of the obtained spectrum at 415 nm is very close to zero (Figure 2 C) and the GP parameter gives relative values only (as pointed out further on), that is, even if biased, one only has to be consistent within one experimental study.

The DOPC and SM:Chol GUVs mimicked an Ld and Lo lipid environment, respectively. This was observed by the blue-shifted fluorescence emission in the case of the Lo environment (SM:Chol). The observed spectra are very similar to the reported spectra of C-Laurdan in these membranes.[12–13] From these spectra, we calculated the GP values for each image pixel [Eq. (1)] with a sufficient amount of fluorescence signal using the direct sampling option of our custom Fiji plug-in (see Materials and Methods section and Figures S1 and S2 in the Supporting Information, SI). The GP values were very homogeneously distributed over the GUVs (Figures 2 D and 2 E) with a fairly disordered (GP≈−0.55) and ordered (GP≈0.5) environment for the DOPC and SM:Chol GUVs, respectively (Figure 2 F). The GP values are close to values previously reported for C-Laurdan in liposomes of the same compositions,[12, 21] and correlated as expected with changing the molecular order or lipid packing of the GUVs by varying the lipid content (DOPC, DOPC:Chol (9:1). DOPC:Chol (4:1) and POPC; Figure S3). For GUVs of ternary lipid mixtures of unsaturated DOPC, saturated SM and cholesterol, the spectral GP image analysis reflected the expected phase separation into Ld and Lo domains on a single GUV (Figure 2 G –J), with the GP values of the Lo and Ld environments depending on the ratio of the lipid mixture (Figure S3).

### 2.2. Curve Fitting: Increasing the Accuracy of GP Imaging

**Figure 3:**
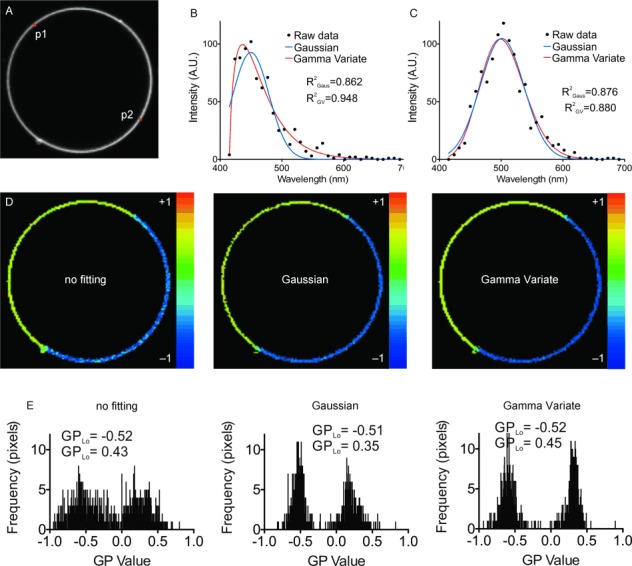
Increasing the accuracy of spectral GP imaging using curve fitting. A) Fluorescence intensity Z-projection image (i.e. addition over the whole spectral image stack, 30 μm x 30 μm) of a representative C-Laurdan-labeled phase-separated GUV (DOPC:SM:Chol, 2:2:1). B,C) Exemplary fluorescence emission spectra taken at pixels p1 (B, Lo phase) and p2 (C, Ld phase) marked in image A with raw data (black dots), Gaussian fit (blue line), and Gamma Variate fit (red line) (fit quality values of the coefficient of determination *R*^2^ as labeled). Note that the spectrum of the Lo phase is better described by a Gamma Variate fit. D) GP image of the GUV of panel A following analysis using direct sampling (non-fitting, left), Gaussian fitting (middle) and Gamma Variate fitting (right). All the analysis methods reveal a phase separation; however, the GP image following fitting is less noisy. E) Histograms of all GP values extracted from the images of panel D as labeled, showing that the GP histograms from the curve fitting (especially the Gamma Variate) are more discrete.

For the calculation of the GP values [Eq. (1)] in Figure 2, we used a direct sampling approach, that is, the intensity values *I*_R_ and *I*_B_ were approximated from the image slices registering fluorescence emission for the wavelength ranges most closely matching *λ*_Ld_ and *λ*_Lo_, respectively (see Materials and Methods). To be comparable to previous studies, we verified that the use of wavelength intervals (as done in previous studies), instead of two discrete wavelengths (as done here), yielded similar results for the GP values (Figure S4). We now anticipated increasing the accuracy of determining *I*_R_, *I*_B_ and thus the GP values by employing information from the whole emission spectrum, that is, from all data points. For this, we fit a Gaussian or a Gamma Variate distribution to the emission spectrum captured for each pixel, as shown in Figures 3 A –C for C-Laurdan in phase-separated GUVs. From the fit, the values of *I*_R_ and *I*_B_ can be determined with arbitrary wavelength accuracy. In addition, extracting values from the fits, rather than just directly sampling the raw data, has a de-noising effect. The GP images determined for the curve fitting are visibly de-noised compared to the direct sampling analysis (Figure 3 D). This becomes even more obvious when comparing the distribution of GP values over images of GUVs from the direct sampling (non-fitting) with that of the curve-fitting analysis (Figure 3 E). While the GP histograms generated from each of the approaches are peaking at similar values, the GP histograms from the curve fitting are more discrete. This is a natural consequence of having used the whole spectrum to infer the fluorescence value rather than sampling the raw data, and is an improvement over the existing GP analysis methods, which can often require significant smoothing prior to GP calculation.[22]

In addition, we experienced that the Gamma Variate fitted the C-Laurdan spectra much better than the Gaussian distribution, especially when the spectrum at that pixel was skewed, as is often the case in the ordered vesicle phase (Figure 3 B). This is also revealed in the average values of the coefficient of determination (*R*^2^=0.86 for Gaussian and 0.94 for Gamma Variate fitting, *p*<0.05, Student′s t-test, *n*=5), which we have used for checking the accuracy of the fits, and whose value is one for a perfect fit and decreases for increasingly inaccurate fitting. As a consequence, the distribution of GP values following the Gamma Variate fitting is slightly more discrete than that resulting from the Gaussian fits (Figure 3 E), especially for the ordered phase. The Gaussian fitting was, however, much quicker (≈tenfold), and is thus a good compromise between accuracy and analysis speed.

### 2.3. Spectral GP Imaging of Cell-Derived Model Membranes

**Figure 4:**
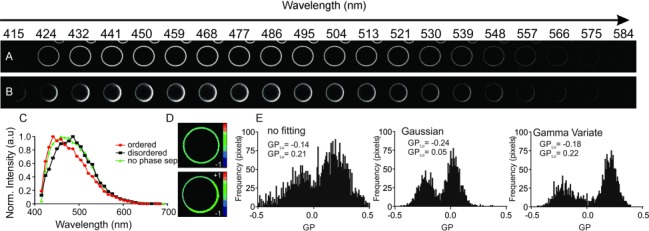
Spectral GP imaging of cell-derived GPMVs. A,B) Simultaneously recorded 10 μm x 10 μm large fluorescence images for 20 different 8.9 nm-wide spectral wavelength windows between 415 and 584 nm of the equatorial plane of representative non-phase-separated (A) and phase-separated (B) GPMVs derived from live RBL cells and labeled with C-Laurdan. The differences in emission maxima are barely visible for the two phases. C) Representative fluorescence emission spectra of C-Laurdan in a single pixel of the recorded image stacks for the non-phase separated GPMVs (green), and the disordered (black, more red-shifted fluorescence) and ordered (red) phases of the phase-separated GPMVs. D) Final GP images (size 10 μm x 10 μm) of the non-phase-separated (top) and phase-separated (bottom) GPMVs. E) Histograms of GP values following direct sampling (non-fitting, left), Gaussian fitting (middle) and Gamma Variate fitting (right) indicating the accuracy with which minute changes in lipid packing can be observed using spectral imaging in combination with curve fitting.

Much attention in the field of biophysical membrane research has been given to the investigation of cell-derived vesicles such as GPMVs.[23] GUVs with only a mixture of a few lipids are limited in complexity when compared to a cellular plasma membrane with its high diversity in lipids and proteins,[24] which is not the case for GPMVs, since they are directly generated from cellular plasma membrane.[23] Despite the increased molecular complexity, GPMVs can also show the coexistence of more ordered and disordered phases,[17, 23] although with a much lower difference in molecular order or GP values between the phases compared to GUVs.[24] Figure 4 depicts how this minute difference in lipid packing is accurately exposed by spectral GP imaging, taking GPMVs derived from RBL (rat basophilic leukaemia) cells as an example. Figure 4 shows the results of our GP analysis for a non-phase-separated and a phase-separated GPMV. As expected:[8, 25] 1) the shift in wavelengths and GP values between ordered and disordered phase is much less pronounced than in GUVs (ordered: GP≈0.2 versus ≈0.4 in GUVs, and disordered: GP≈−0.15 versus ≈−0.5 in GUVs, compare Figures 2 and 3),[17] and 2) the lipid packing in non-phase-separated GPMVs (GP≈−0.05) is in-between that of the Lo and Ld environments of the phase-separated GPMVs (Figure 4 C, D). These minor differences in lipid packing or GP values are more accurately explored using spectral GP imaging in combination with our curve-fitting approach, as highlighted in Figure 4 E. Please note that (as for the results from the GUV experiments) the Gamma Variate fitting gives more reliable results than the Gaussian analysis; especially the GP value of the Lo environment is more closer to the value of the conventional direct sampling analysis.

### 2.4. Spectral GP Imaging of the Live-Cell Plasma Membrane

Next, we applied spectral GP imaging to live-cell plasma membranes. Unfortunately, C-Laurdan is strongly internalized in living cells,[7] despite the cell labeling on ice, which makes the determination of GP maps less accurate (Figure S5). Fortunately, spectral imaging allows the straightforward implementation of other polarity-sensitive probes with different emission spectra as it obviates probe-specific filter sets. Here, we used the membrane-dye Di-4-ANEPPDHQ,[5, 14] which shows significantly less internalization in living cells.[7] Its fluorescence emission was excited at 488 nm and recorded between 495 and 691 nm. For this dye, we picked *λ*_Ld_=565 nm and *λ*_Lo_=610 nm, following previous work[7] (see Materials and Methods). Spectral imaging using this probe in GUVs and GPMVs yielded a GP value of −0.2 (with an emission maximum of 610 nm) in liquid-disordered DOPC GUVs and 0.5 (with an emission maximum of 565 nm) in liquid-ordered SM:Chol GUVs (Figure S6). However, the selection of *λ*_Ld_ and *λ*_Lo_ for Di-4-ANEPPDHQ is rather arbitrary, with another choice resulting in other GP values[14] (see Materials and Methods). Also, the GP values for C-Laurdan and Di-4-ANEPPDHQ are different due to different dependencies of their respective fluorescence emission on lipid packing (note that the spectral shift between the Lo and Ld conditions is much less pronounced than for C-Laurdan). In a nutshell, the GP values are relative values, and one only has to be consistent within one experimental study.

**Figure 5:**
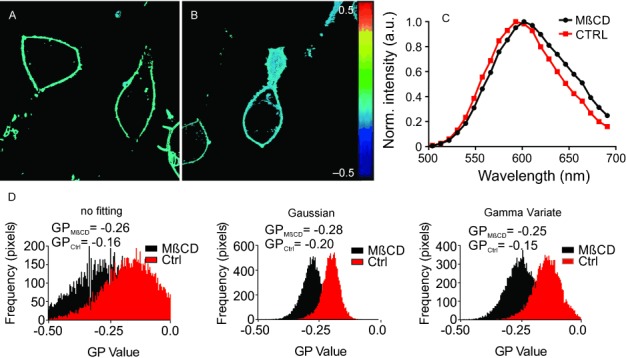
Spectral GP imaging of live RBL cells. A,B) GP images (40 μm x 40 μm) of the equatorial plane of live RBL cells using the environment-sensitive membrane dye Di-4-ANEPPDHQ, without (A) and with (B) MβCD treatment. C) Representative fluorescence emission spectra of Di-4-ANEPPDHQ at a single pixel of the recorded image stacks for the untreated (red) and MβCD-treated cells (black). D) Histograms of GP values following the different analysis routines (as labeled) for the untreated (ctrl, red) and MβCD treated (black) experiments.

Generally, the sensitivity of Di-4-ANEPPDHQ on changes in lipid packing is lower than that of C-Laurdan,[7] which makes an accurate determination of the intensity values *I*_R_ and *I*_B_ even more essential. Figure 5 shows the spectral GP image analysis of Di-4-ANEPPDHQ labeled live RLB cells, with an average GP value of ≈−0.16. As expected,[26] lipid packing is further reduced (GP≈−0.26) after cholesterol depletion of the cellular plasma membrane using treatment with methyl-beta cyclodextrin (MβCD). Again, the GP histograms following the curve-fitting analysis are much more discrete, allowing for a much better distinction of lipid packing between the two conditions (Figure 5 D). In contrast to C-Laurdan, for Di-4-ANEPPDHQ the Gaussian fitting produces the most discrete distribution of GP values, but again with slightly different peak values compared to the Gamma Variate fitting and direct sampling approach. As mentioned before, the GP values are relative values, and one has to be consistent within one experimental study, here also with respect to the analysis procedure.

## 3. Conclusions

Lipid packing is considered an important organizational and functional aspect of the cellular plasma membrane.[1–2] Accurate measurements of lipid packing of model and cellular membranes allow gaining insights into physical chemistry properties of the membranes and relating these to their bioactivity. Fluorescent polarity-sensitive probes in combination with generalized polarization (GP) analysis are often used to quantify the lipid packing. While the determination of GP values from a fluorescence spectrophotometer is straightforward and accurate, the imaging of the spatial variation in lipid packing is more challenging. Here, we approach this challenge by spectral GP imaging using the possibility of state-of-the-art confocal microscopes to simultaneously detect the spectrally dispersed fluorescence signal on different detectors. The resulting image stack (where each slice represents the fluorescence signal detected within a small wavelength interval) enables the instantaneous setup of fluorescence emission spectra for each image pixel. The spectral dispersion of the signal can either be achieved using a prism and a 32-channel GaAsP line-array detector, as in this case, or gratings or acoustic–optical elements in conjunction with an array of multiple detectors. Using a custom-designed Fiji/ImageJ plug-in, we showed that this spectral GP imaging approach revealed a straightforward way for observing spatial heterogeneity in lipid packing of model as well as cellular membranes. Specifically, we demonstrated that heterogeneity in lipid packing can more accurately be determined by using the whole spectrum to infer fluorescence values at discrete wavelengths for GP calculation rather than using raw data. More importantly, due to its ability to yield a single pixel spectrum, we believe that spectral imaging will in the future enable us to overcome the two-wavelength GP calculation limitation and to use the whole spectrum to understand the dipolar relaxation phenomenon in the context of biological membrane ordering.

## Experimental Section

### Materials

We used the following lipids and fluorescent lipid analogs: 1,2-dioleoyl-sn-glycero-3-phosphatidylcholine (DOPC), 1-palmitoyl-2-oleoyl-*sn*-glycero-3-phosphocholine (POPC), N-stearoyl-d-erythro-sphingosylphosphorylcholine (SM), and 1,2-dipalmitoyl-sn-glycero-3-phosphocholine (DPPC) (Avanti Polar Lipids), C-Laurdan (2pprobes), Di-4-ANEPPDHQ (Invitrogen), and cholesterol, N-ethylmaleimide (NEM), β-cyclodextrin, cellular media (RPMI, FCS, MEM) and dithiothreitol (Sigma).

### Giant Unilamellar Vesicle Preparation

GUVs were prepared as previously described.[17] Briefly, 1 mg mL^−1^ lipid solution was prepared in chloroform. Then, 5 μL of this solution were dried onto two parallel platinum wires mounted in a custom-built GUV Teflon chamber.[24] A 300 mm sucrose solution was added to the chamber and a 10 Hz current was applied to the wires for an hour.[24] GUV preparation of SM:Chol, DPPC:Chol or DOPC:SM:Chol mixtures was performed at 70 °C above the respective lipid transition temperature. Other GUVs (such as pure DOPC) were formed at room temperature.

### Cell Maintenance

RBL cells were grown in 60 % RPMI, 30 % MEM and 10 % FCS medium. They were seeded out two days before the experiments so that they would reach 70 % confluence on the day of the experiment.

### Giant Plasma Membrane Vesicle Preparation

GPMVs were prepared as previously described.[17] Briefly, cells seeded out on a 60 mm petri dish (≈ 70 % confluent) were washed twice with a GPMV buffer (150 mm NaCl, 10 mm Hepes, 2 mm CaCl2, pH 7.4). 2 mL of GPMV buffer was added to the cells. 25 mm PFA and 10 mm DTT (final concentrations) were added in the GPMV buffer. The cells were incubated for 2 h at 37 °C. Then, GPMVs were collected by pipeting out the supernatant.

### Cholesterol Modulation

For cholesterol removal, 0.12 g methyl-beta cyclodextrin was dissolved in 10 mL MEM (10 mm). Then the cells seeded out on 8-well glass bottom Ibidi slide (#1.5) chambers were incubated with 250 μL/well of this suspension for 30 min at 37 °C. Cells were washed with phosphate-buffered saline (PBS) a few times before labeling.

### Membrane Labeling with C-Laurdan and Di-4-ANEPPDHQ

The GUVs and GPMVs were spiked with C-Laurdan or Di-4-ANEPPDHQ at a final concentration of 0.4 μm at room temperature. The cells were incubated with a 0.4 μm probe solution in PBS for 5 min on ice to reduce the internalization of the probes. Then, the cells were washed a few times with PBS. 250 μL of cell media without phenol red and serum was added onto the cells for imaging. Samples were imaged in 8-well glass bottom Ibidi chambers (#1.5).

### Confocal Spectral Imaging

Spectral imaging of the different membrane samples was performed on a Zeiss LSM 780 confocal microscope equipped with a 32-channel GaAsP detector array. Laser light at 405 and 488 nm was selected for fluorescence excitation of C-Laurdan and Di-4-ANEPPDHQ, respectively. The lambda detection range was set between 415 and 691 nm for C-Laurdan, and between 495 and 691 nm for Di-4-ANEPPDHQ (Figure S1). The wavelengths 415 and 691 nm were the ultimate limits of our detector. Despite the fact that wavelength intervals of down to 4 nm could be chosen for the individual detection channels, we have set these intervals to 8.9 nm, which allowed the simultaneous coverage of the whole spectrum with the 32 detection channels (Figure S1). The images were saved in .lsm file format and then analyzed by using a custom plug-in compatible with Fiji/ImageJ, as described further on.

### Obtaining Spectra from the Spectral Images

The spectra for each image pixel were obtained from the intensity values of the 32 different detection channels by using the ImageJ plug-in “Stacks-T functions-Intensity vs. Time Monitor”. We usually applied the plugin only on pixels within a region of interest of the acquired images. The background signal was determined by applying the same plugin on a dark region (of the same size) of the image, and subtracted from the signal from the region of interest.

### Definition of *λ*_Ld_ and λ_Lo_

For the calculation of the GP values [Eq. (1)], one has to define the wavelengths *λ*_Ld_ and *λ*_Lo_ of maximum emission of a probe in a reference liquid-disordered (Ld) and liquid-ordered (Lo) membrane environment. Following the literature, for C-Laurdan we have chosen *λ*_Ld_=490 nm and *λ*_Lo_=440 nm.[27] For the other probe, Di-4-ANEPPDHQ, we have chosen *λ*_Ld_=605 nm and *λ*_Lo_=565 nm, as defined in a previous work.[7] However, the wavelength selection for Di-4-ANEPPDHQ is rather arbitrary, and a choice of a more blue-shifted *λ*_Lo_ and a more red-shifted *λ*_Ld_ may increase the GP contrast. Therefore, GP values are relative values; that is, the GP values determined via C-Laurdan are different from those determined via Di-4-ANEPPDHQ, or when choosing other wavelengths *λ*_Ld_ and *λ*_Lo_. One only has to be consistent within one experimental study.

### GP Calculation Using the Plug-in

A Java Fiji plug-in was developed for an efficient analysis of the acquired spectral data (plug-in is available at https://github.com/dwaithe/GP-plugin). This plugin facilitates calculation of the GP equation, with direct sampling of the original wavelength intervals or through fitting of the entire available spectrum at each pixel using a curve-fitting algorithm. In any case, the plug-in determined discrete intensity values, *I*_R_ and *I*_B_, for the detected signal at *λ*_Ld_ and *λ*_Lo_, and calculated GP values according to Equation (1).

As a preprocessing step for both techniques, the spectral image stacks are integrated via Z-projection and then masked using a user-defined threshold on an 8-bit scale (0–255). This step creates a mask that is used to restrict subsequent analysis to areas positive for fluorescence above the background only. Furthermore, an optional step for the user is to include an average background subtraction correction in the pre-processing. If this option is selected, the background signal intensity is calculated from the dark areas of each image of the whole spectral stack (individual slices), and this value is then subtracted from each slice in the areas selected for the positive signal. This measure is included to correct minor variations in the background signal levels between slices.

Every slice in the image stack represents a certain wavelength *λ*, usually the central wavelength in the corresponding 8.9 nm-wide wavelength interval, and values of intensity *I*_*λ*_ at different wavelengths *λ*, that is, the emission spectra are obtained from the pre-processed image stack for each pixel. At this point, correction factors can be included, accounting for a potential difference in sensitivity between the different spectral detection channels (which was not required in our case, since the microscope supplier guaranteed pre-normalization of the different channels) (Figure S2). From this *I*_*λ*_ distribution, the plug-in determines *I*_R_ and *I*_B_ for each pixel in the masked image by: 1) selecting *I*_*λ*_ values for a wavelength range most closely to *λ*_Ld_ and *λ*_Lo_ (direct sampling), or 2) *I*_R_ and *I*_B_ modelling using curve-fitting, where the ImageJ “CurveFitter API” is applied to fit either the Gamma Variate or the Gaussian no-offset distribution to the *I*_***λ***_ distribution. To judge the quality of the fit, the *R*^2^ measure is extracted and only pixels above a user-defined noise tolerance are included (0.0–1.0, 1.0 being the best fit). Upon successful fitting, inference is performed on the generated distributions to precisely (with 1 nm precision) determine *λ*_Ld_ and *λ*_Lo_ and from that obtain *I*_R_ and *I*_B_. Before final calculation of the GP values using Equation (1), the values of *I*_R_ and *I*_B_ are normalized through division by 255.

Application of the above calculation produces a spatial GP map representing the GP value for each pixel of the image. The final stage of the plugin calculates and outputs a histogram of the GP map and applies a custom look-up-table (LUT) to the data. Along with the histogram and GP map, the plug-in also outputs general histogram statistics along with median values for the positive and negative phases of the GP distribution, the selected *λ*_Ld_ and *λ*_Lo_ input wavelengths (either from the original input stack or from inference), the calculated mask image, and also, for the curve-fitting, the goodness of fit image, where each pixel is labeled with its *R*^2^ value.

## Supporting Information

As a service to our authors and readers, this journal provides supporting information supplied by the authors. Such materials are peer reviewed and may be re-organized for online delivery, but are not copy-edited or typeset. Technical support issues arising from supporting information (other than missing files) should be addressed to the authors.

miscellaneous_information
