# Supplementary material for: Spectral Imaging to Measure Heterogeneity in Membrane Lipid Packing
Source: Chemphyschem. 2015 Mar 5;16(7):1387–94. doi: 10.1002/cphc.201402794 (PMC4539592; doi:10.1002/cphc.201402794)
Supplement: Supplementary file 1 — miscellaneous_information [file cphc0016-1387-sd1.pdf]

CHEMPHYSCHEM

Supporting Information

Spectral Imaging to Measure Heterogeneity in Membrane Lipid Packing

Erdinc Sezgin,* Dominic Waithe, Jorge Bernardino de la Serna, and Christian Eggeling*[a]

cphc_201402794_sm_miscellaneous_information.pdf

Supplementary Information

Supplementary Figures

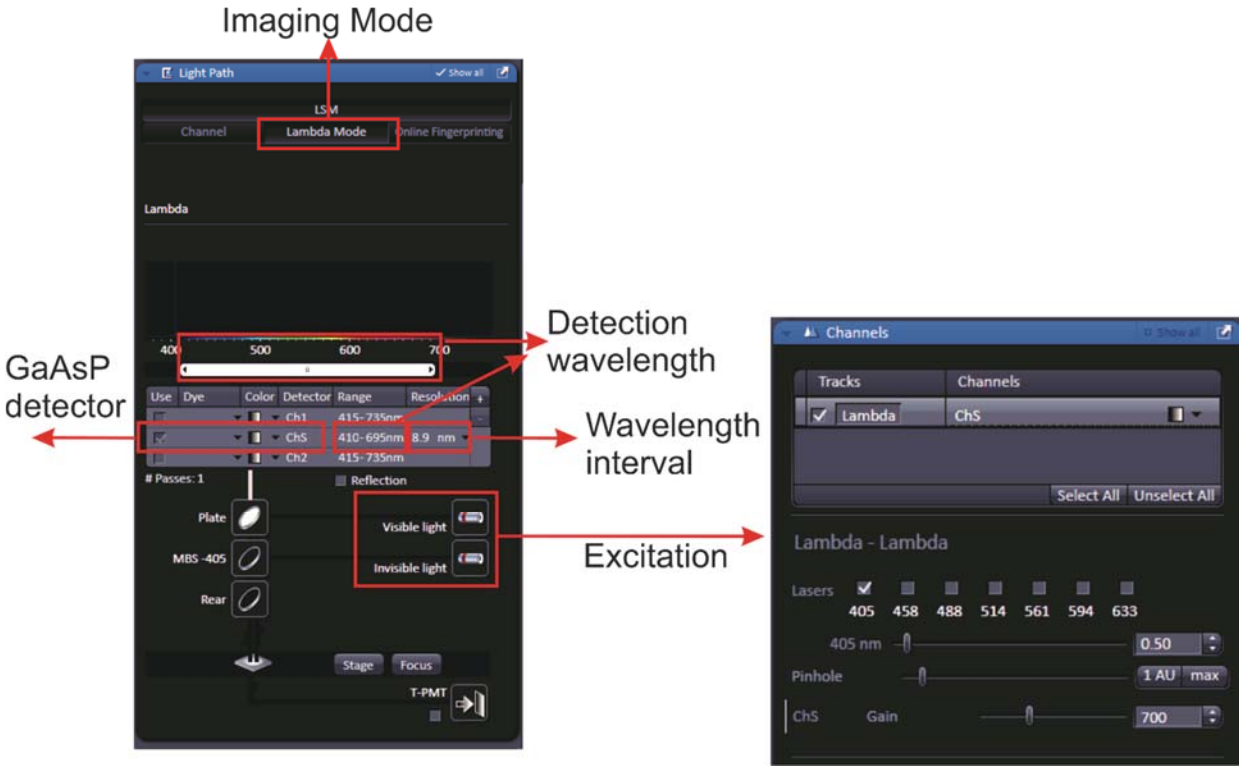

Figure S1. Screenshot of settings in Zen software of the Zeiss 780 confocal microscope for spectral imaging.

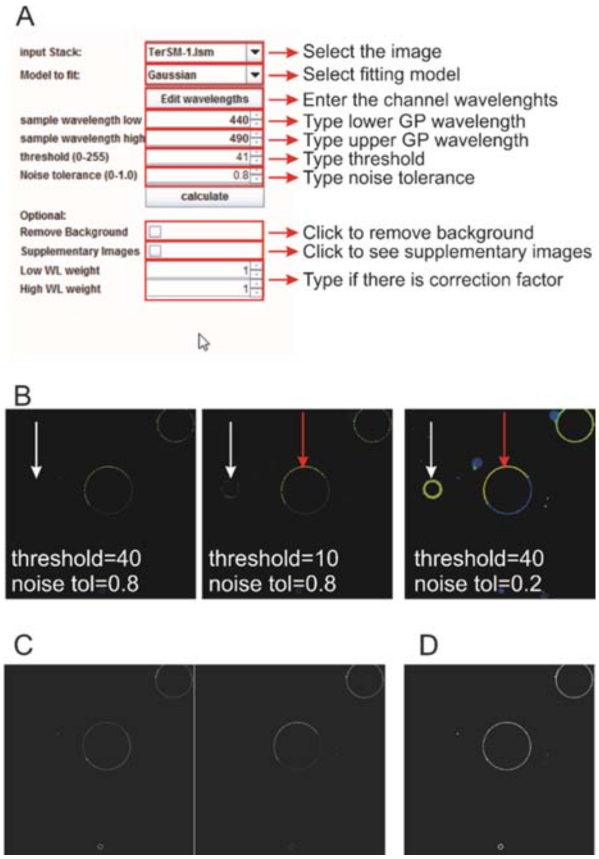

Figure S2. Fiji plug-in. (A) Screenshot of plug-in user interface with inputs as labeled. The plug-in automatically obtains the wavelength information of the image stack from the .lsm or .czi files (as well as from other image file formats). Input parameters are: Analysis type (direct sampling, Gaussian or Gamma Variate fitting), λ_{Ld} and λ_{Lo} (lower and upper GP wavelength), signal threshold (discriminating between real fluorescence (to be analyzed) and unwanted background signal (not analyzed)), and noise tolerance (represents the "R^2" parameter used during fitting; acceptable range of fit quality with "1" being the perfect fit). Further, the user can choose whether to include the background removal, supplementary data, and whether there are any correction factors to be applied (see below). (B) Effect of threshold and noise tolerance: A too high threshold suppresses signal (white arrow), while a too low noise tolerance results in unwanted features in the image. Further, low noise tolerance will increase the signal (red arrow) as it includes the pixels with lower fit quality. (C) Supplementary ordered/disordered channel images and (D) binary mask with pixels which are used for GP calculation created using selected threshold value. 'Remove Background' option subtracts from each channel in the image-stack the average background pixel value of each respective channel (background region is taken as the complement to binary mask image) prior to GP calculation. Low WL (wavelength) and High WL options specify additional weightings to be applied to the intensities measured at the lower and higher GP wavelengths. The wavelength weightings allow non-normalised detectors to be corrected so that GP calculation can be compared across different acquisition equipment. The Zeiss GaAsP detector is pre-normalised for non-linear detection efficiency across the spectrum and so further weightings were not required in this study. Furthermore, background noise was found to be low throughout this investigation and so background subtraction was not required in this case either.

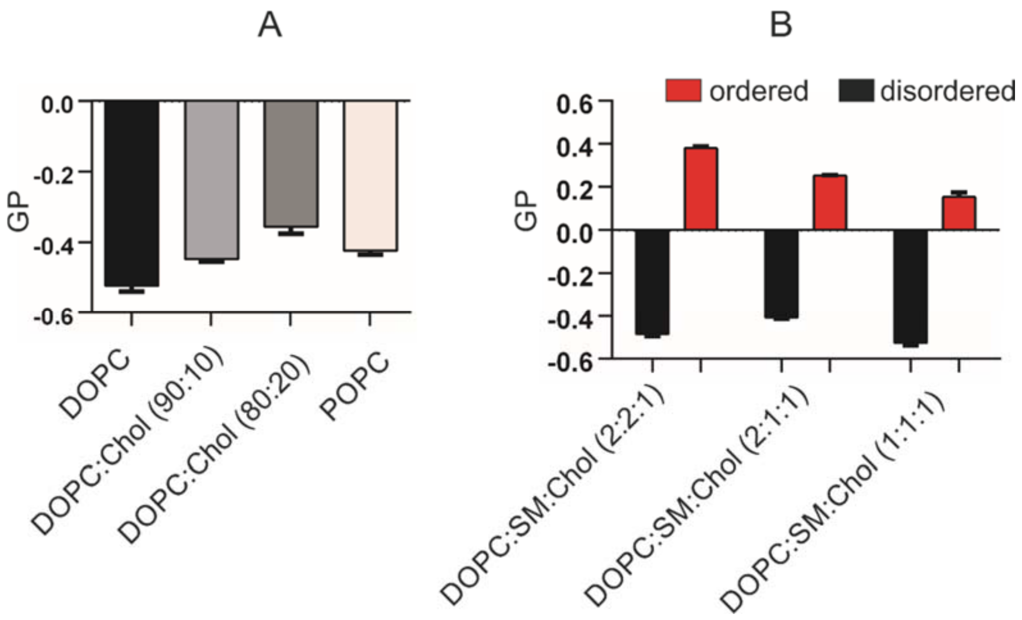

Figure S3. Results of spectral GP image analysis of GUVs with different lipid mixtures as labeled. (A) One-component GUVs: Effect on cholesterol (DOPC with different amounts of cholesterol) and effect of lipid acyl chain (POPC versus DOPC). Note the increase in lipid packing for POPC (longer acyl chain) and with increasing amounts of cholesterol. (B) Phase-separated GUVs: Changes of GP with composition of ternary DOPC/SM/Cholesterol mixture (as labeled). Note the decrease in lipid packing of the ordered phase with decreasing relative amount of DOPC and SM.

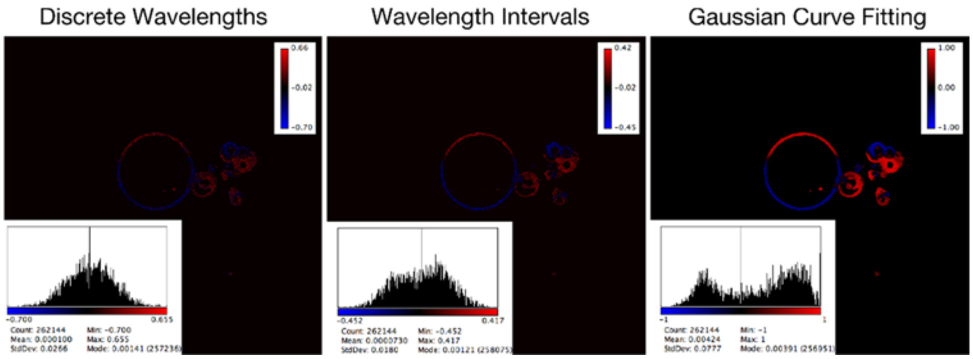

Figure S4. Comparison of using two discrete wavelengths (565 nm and 605 nm), two wavelength intervals (550-580 nm and 590-620 nm) and Gaussian curve fitting for GP calculation: Representative GP images and histograms of GP values from pixels with fluorescence signal for DOPC:DPPC:Chol (2:2:1) vesicles doped with Di-4-ANEPPDHQ. Gaussian curve fitting gives better resolution than discrete wavelengths and wavelength intervals which yield similar results.

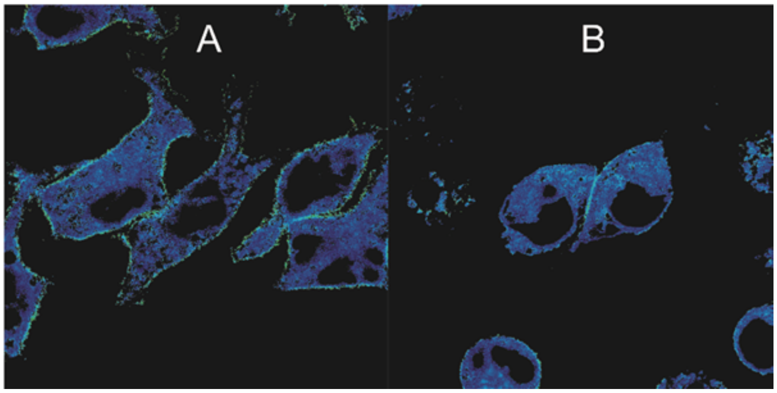

Figure S5. Spectral GP imaging of live RBL cell plasma membranes using C-Laurdan. Equatorial GP image of (A) control cells and (B) cholesterol depleted cells. The strong internalization of C-Laurdan makes an accurate determination of GP values less optimal.

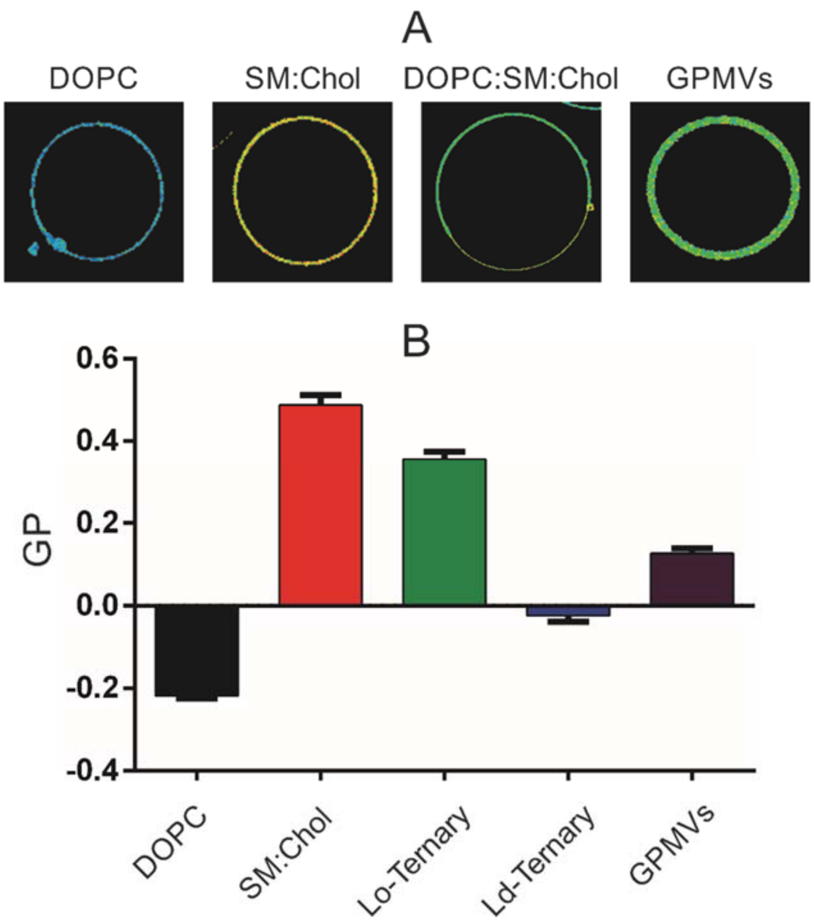

Figure S6. Spectral GP imaging of GUVs using Di-4-ANEPPDHQ. (A) GP images (30 µm x 30 µm) of GUVs of different lipid mixtures as labeled. (B) Quantification of average GP values and standard errors of the different vesicles of A.
